# Src tyrosine kinase augments taxotere-induced apoptosis through enhanced expression and phosphorylation of Bcl-2

**DOI:** 10.1038/sj.bjc.6600080

**Published:** 2002-02-01

**Authors:** V Boudny, S Nakano

**Affiliations:** Department of Medicine and Biosystemic Science, Graduate School of Medicine, Kyushu University, 3-1-1 Maidashi, Higashi-Ku, Fukuoka, Fukuoka 812-8582, Japan

**Keywords:** v-Src, taxotere (docetaxel), Bcl-2 phosphorylation, apoptosis

## Abstract

Activation of Src, which has an intrinsic protein tyrosine kinase activity, has been demonstrated in many human tumours, such as colorectal and breast cancers, and is closely associated with the pathogenesis and metastatic potential of these cancers. In this study, we have examined the effect of activated Src on the sensitivity to taxotere, an anticancer drug targeting microtubules, using v-*src*-transfected HAG-1 human gall bladder epithelial cells. As compared with parental HAG-1 cell line, v-*src*-transfected HAG/src3-1 cells became 5.9 and 7.0-fold sensitive to taxotere for 2 and 24-h exposure, respectively. By contrast, HAG-1 cells transfected with activated Ras, which acts downstream of Src, acquired approximately 2.5∼4.8-fold taxotere resistance. The taxotere sensitivity in HAG/src3-1 cells was reversed, if not completely, by herbimycin A, a specific inhibitor of Src family protein tyrosine kinase, indicating that Src protein tyrosine kinase augments sensitivity to taxotere. Treatment of HAG/src3-1 cells with taxotere resulted in phosphorylation of Bcl-2 and subsequent induction of apoptotic cell death, whereas neither Bcl-2 phosphorylation nor apoptosis occurred in parental or c-H-*ras*-transfected HAG-1 cells. Interestingly, the Bcl-2 protein is overexpressed in v-*src*-transfected cell line, compared to those in parental or Ras-transfected cell line. Treatment of HAG/src3-1 cells with herbimycin A significantly reduced the expression and phosphorylation of Bcl-2, and abrogated taxotere-induced apoptosis, suggesting a potential role for Src protein tyrosine kinase in the taxotere-induced apoptotic events. H-7, a protein kinase C inhibitor and wortmannin, a phosphatidylinositol-3 kinase (PI-3 kinase) inhibitor, neither altered taxotere sensitivity nor inhibited taxotere-induced apoptosis in these cells. These data indicate that the ability of activated Src to increase taxotere sensitivity would be mediated by apoptotic events occurring through Src to downstream signal transduction pathways toward Bcl-2 phosphorylation, but not by activated Ras, PI-3 kinase or protein kinase C.

*British Journal of Cancer* (2002) **86**, 463–469. DOI: 10.1038/sj/bjc/6600080 www.bjcancer.com

© 2002 The Cancer Research Campaign

## 

Taxanes, the novel chemotherapeutic drugs, have unique mechanisms of action that promote tubulin polymerization as well as the formation and stabilization of microtubules, thus blocking cell cycle at the metaphase to anaphase transition (Schiff *et al*, 1979; Ringel and Horwitz, 1991; Horwitz, 1992). Although the arrest of cells in the G2-M phase of the cell cycle correlates with taxane-induced apoptosis (Woods *et al*, 1995), the precise mechanism of apoptotic action of taxanes still remains unclear. The phosphorylation of Bcl-2, a prototype of a related group of proteins implicated in regulating apototic cell death (Reed, 1994), has been reported as a potential cause of taxane-induced apoptosis (Haldar *et al*, 1995, 1996, 1997). Certain members of the Bcl-2 family are proapoptotic (i.e. Bcl-X_s_, Bad, Bax), while others are antiapoptotic (i.e. Bcl-2, Bcl-X_L_, Mcl-1) (Oltvai *et al*, 1993; Reed, 1997). Several studies have demonstrated that Bcl-2 phosphorylation can be specifically induced by drugs targeting microtubules, whereas this effect is not seen by DNA-damaging agents (Haldar *et al*, 1997). The Bcl-2 phosphorylation at serine residues leads to loss of Bcl-2 anti-apoptotic function (Haldar *et al*, 1995) by inhibiting its binding to the proapoptotic Bax protein (Haldar *et al*, 1996). Thus, it is suggested that prevention of depolymerization of cellular microtubules by taxanes causes phosphorylation of Bcl-2, thereby abrogating the normal anti-apoptotic function of Bcl-2 and initiating the apoptotic program in the cycling cancer cells.

Certain oncogenes, such as v-*src*, activate both mitogenic and survival signaling pathways. v-Src is a mutationally activated form of the non-receptor tyrosine kinase c-Src, and c-Src has been shown to be activated frequently in human cancers, such as breast (Hennipman *et al*, 1989; Ottenhoff-Kalff *et al*, 1992), colon (Bolen *et al*, 1987; Cartwright *et al*, 1990; Talamonti *et al*, 1993), skin (Barnekow *et al*, 1989), bladder (Fanning *et al*, 1992) and pancreas cancers (Lutz *et al*, 1998). Specifically, c-Src has been found to be highly activated in colon cancer metastasized to the liver (Mao *et al*, 1997) and mutation in the regulatory domain of c-Src has been reported as a mechanism of Src activation in human colon cancer (Irby *et al*, 1999). Although a number of signal transduction pathways toward Bcl-2 phosphorylation have been reported as an apoptotic mechanism induced by taxanes (Wang *et al*, 1999), the role of Src in these processes has yet to be determined. Src transduces a variety of signals to downstream signal transduction cascades including Ras (Brown and Cooper, 1996). Thus, activation of Src may potentially affect the apoptotic events induced by taxanes, because several lines of evidence suggest that apoptosis induced by those microtubule-targeting agents require the Ras/c-Raf-1/Bcl-2 pathway (Blagosklonny *et al*, 1996, 1997, 1999). However, the direct association between Src, Bcl-2 phosphorylation, and the sensitivity to taxanes has not been studied.

Recently, we have studied whether activated Src induces chemoresistance by evaluating alterations of drug sensitivity in human HAG-1 gall bladder epithelial cells transfected with v-*src* oncogene and determined the mechanism of drug resistance. We have found that v-*src* induces resistance to cisplatin (CDDP) through activation of the repair of CDDP-induced DNA damage (Masumoto *et al*, 1999). In continuing these studies, we have recently found that v-Src induces significant sensitivity to taxotere, a semi-synthetic taxol analogue. In this report, we have investigated the cellular and molecular mechanism(s) whereby Src induces taxotere sensitivity, with special reference to taxotere-induced apoptosis and Bcl-2 phosphorylation, using v-*src*-transfected HAG-1 human epithelial cells. We found that Src tyrosine kinase augments taxotere-induced apoptosis by enhancing Bcl-2 expression and phosphorylation.

## MATERIALS AND METHODS

### Cells and cultures and chemicals

HAG-1 is a human epithelial cell line derived from a moderately differentiated adenocarcinoma of the gallbladder (Nakano *et al*, 1994). No mutations and amplifications of H-, K-, or N-*ras* genes have been detected in this cell line. The HAG-1 cells do not grow in soft agar and have remained non-tumorigenic in nude mice. HAG/ras5-1 cells were obtained by transfecting HAG-1 parental cells with activated c-H-*ras*. The H-*ras-*transfected clone cannot grow in soft agar despite expression of activated p21^*ras*^ oncoprotein, whereas HAG/src3-1 cells, obtained by transfection of the pSV2/v-*src* into HAG-1 cells, express p60^v-*src*^ protein, grow in soft agar and are highly tumorigenic (Tatsumoto *et al*, 1995). Individual cells were cultured at 37°C in Dulbecco's minimum essential medium (DMEM, Nissui, Tokyo, Japan) supplemented with 10% heat-inactivated foetal bovine serum (FBS, Gibco, Grand Island, NY, USA) in a humidified atmosphere of 5% CO_2_ and 95% air.

Wortmannin and Herbimycin A (HA) were obtained from Wako Chemicals (Osaka, Japan). H-7 [1-(5-isoquinolinylsulfonyl)-2-methylpiperazine] were obtained from Sigma (St Louis, MO, USA). H-7 was dissolved in distilled water as 10 mM stock solution and stored at 4°C. Taxotere (Docetaxel) was a kind gift from Rhone-Poulenc Rorer (Tokyo, Japan). Taxotere, HA and wortmannin were dissolved in 100% demethyl sulphoxide (DMSO) as 5000× stock solutions and aliquots were frozen. All solutions were prepared fresh by diluting with DMEM on the day of use. The final concentration of DMSO for all experiments and treatments (including controls, where no drug was added) was maintained at less than 0.02%. These conditions were found to be non-cytotoxic.

### Cell-survival assay

Cells were seeded in triplicate in 4 ml of complete medium into 60-mm tissue culture dishes (Falcon 3002; Oxnard, CA, USA) such that the control cultures did not reach confluence, in order to avoid the influence of density inhibition of cell growth on cytotoxicity. The cells were incubated overnight to allow attachment to the plastic prior to administration of the drug, and exposed to various concentrations of taxotere for 2 or 24 h. Following each taxotere treatment, the cells were washed twice with phosphate buffered saline (PBS, Nissui, Tokyo, Japan) and the medium was replaced with fresh complete medium for an additional 3 days. Then the medium was replaced with complete medium. The cells were continuously cultured after drug treatment for 7 days. Trypsinized cells were counted using a Coulter counter (model ID; Hialeah, FL, USA). The percentage of cell growth for taxotere was calculated by dividing the number of cells in the drug-treated culture by the number of cells in the culture not exposed to the drug. For each cell line, at least five independent experiments with triplicate samples were performed.

### Treatment with Src tyrosine kinase inhibitor

Cells were plated in triplicate in 4 ml of complete medium in 60-mm tissue culture dishes, allowed to attach overnight and then incubated with various concentrations of taxotere for 2-h with or without HA, a well characterized inhibitor of Src family tyrosine kinase (Fukazawa *et al*, 1991). After each treatment, the cells were washed twice with PBS, and the medium was replaced with fresh complete medium with or without HA for an additional 72 h. The cell number was determined on day 7. The percentage of cell growth was compared with that of treatment with the drug alone. For both cell lines, parental HAG-1 and v-*src-*transfected HAG/src3-1, five independent experiments with triplicate samples were performed.

### Treatment with protein kinase C and phosphatidylinositol-3 kinase inhibitors

To examine the role of protein kinase C (PKC) in the cytotoxicity for taxotere, the cells were exposed to various concentrations of taxotere for 2 h with or without H-7, a PKC inhibitor (Hidaka *et al*, 1984). The medium was replaced with fresh complete medium, but H-7 treatment was continued for an additional 72 h. The cell number was determined on day 7. To study the role of phosphatidylinositol-3 kinase (PI-3 kinase) in the cytotoxicity for taxotere, cells were preincubated for 30 min with wortmannin, an inhibitor of PI-3 kinase (Yano *et al*, 1993), immediately before taxotere treatment, and treated with various concentrations of taxotere for 2 h. The number of cells was determined on day 7 after treatment with taxotere. The percentage of cell growth was compared with that of treatment with taxotere alone. For each treatment, three independent experiments with triplicate samples were performed.

### Immunoprecipitation and Western blot analysis

Exponentially growing cells (approximately 5×10^6^ cells) were treated for 24 h with taxotere, then washed twice in ice-cold PBS. At various points in time, floating and trypsinized adherent cells were combined, and cells were lysed for 30 min on ice in lysis buffer containing 50 mM Tris-Cl (pH 7.2), 150 mM NaCl, 2 mM EDTA, 0.1% SDS, 1% sodium deoxycholate, 1% Triton X-100, protein tyrosine phosphatase inhibitor (1 mM Na_3_VO_4_), and protease inhibitors (1 mM phenylmethylsulfonyl fluoride, 20 μg ml^−1^ aprotinin, 20 μg ml^−1^ leupeptin, and 20 μg ml^−1^ pepstatin). After centrifugation at 15 000 r.p.m. at 4°C for 15 min, supernatant proteins were immunoprecipitated using protein G-Sepharose 4B (Sigma, St Louis, MO, USA) preconjugated overnight at 4°C with mouse monoclonal antibody to Bcl-2 (Genosys, Pampisford, Cambridgeshire, UK). The Sepharose beads were isolated by centrifugation at 12 000 r.p.m. for 2 min. The immuno-precipitates were then washed four times with ice-cold lysis buffer, resuspended in SDS–PAGE sample buffer, boiled for 5 min, and pelleted by centrifugation at 12 000 r.p.m. for 2 min. The protein content of the supernatant was determined with the protein assay kit (Bio-Rad, Hercules, CA, USA), according to manufacturer's instructions. Equal amounts of protein from each sample were loaded onto 12% SDS–PAGE and electrophoretically transferred to nitrocellulose membrane (type Hybond ECL; Amersham, Japan). Nonspecific binding on the nitrocellulose membrane was minimized with blocking buffer containing 3% bovine serum albumin (BSA, Sigma, St Louis, MO, USA) in Tris-buffered saline. The proteins were probed with primary antibody (the mouse monoclonal anti-Bcl-2 antibody) for 2 h at room temperature and then with secondary antibody, horse radish peroxidase-conjugated goat anti-mouse IgG antibody (ICN Biomedicals/Cappel, Aurora, OH, USA) for 1 h at room temperature. The protein bands were visualized using an enhanced chemiluminescence system (ECL; Amersham, Japan).

### Taxotere-induced apoptosis by DNA fragmentation assay

Exponentially growing cells were treated with 100 nM taxotere for 24 h, washed twice with PBS, and incubated for an additional 24 h in drug-free complete medium. Floating and trypsinized adherent cells were combined. Low-molecular weight genomic DNA was extracted from ∼5×10^6^ cells by a guanidine thiocyanate method using Sepa Gene Kit (Sanko Junyaku, Tokyo, Japan) and precipitated with isopropanol. The final concentration of DNA was determined by UV absorbance at 260 nm (Beckman DU-530 spectrophotometer, Fullerton, CA, USA). Approximately 20 μg of DNA were loaded on each lane of 2% agarose (Sigma, St Louis, MO, USA) gels. DNA was stained with ethidium bromide (1 μg ml^−1^) and visualized under electronic UV transilluminator (Ultra-Lum, Carson, CA, USA) using a CCD black-and-white video camera module (Sony, Tokyo, Japan).

### Intracellular taxotere content

Exponentially growing cells (∼1×10^7^ cells) were treated with 55.0 nM taxotere for 2-h (IC_50_ values for parental HAG-1 cells), and then washed three times with ice-cold PBS. After trypsinization, cells were dissolved in 1 ml of ice-cold PBS, lysed by sonication (Branson Ultrasonics, Danbury, CT, USA), and centrifuged (Himac centrifuge; Hitachi, Tokyo, Japan). The supernatants were frozen at −80°C and later analyzed for taxotere concentration by a rapid reversed phase HPLC method as described (Rouini *et al*, 1998). The intracellular concentration was expressed as relative values per 10^6^ cells.

### Statistical analysis

The data were analyzed by the Mann–Whitney *U*-test for statistical significance of the difference between groups. A *P* value of <0.01 was considered to indicate statistical significance.

## RESULTS

### Chemosensitivity profile

To determine whether activated Src PTK (p60^v-*src*^) affects drug cytotoxicity, we examined the taxotere sensitivity in v-*src-*transfected human gall bladder epithelial cells (HAG/src3-1), and compared their IC_50_ values with those of parental HAG-1 cell line. The respective IC_50_ values of taxotere for 2 and 24-h exposures were 55.4±1.86 nM and 5.05±0.25 nM for HAG-1 parental cells, and 9.47±0.32 nM and 0.725±0.017 nM for HAG/src3-1 cells, indicating approximately 6–7-fold increase in sensitivity to taxotere. By contrast, HAG-1 cells transfected with activated H-*ras* (HAG/ras5-1) exhibited the IC_50_ value of 263.7±7.22 nM and 12.7±0.20 nM for 2 and 24-h exposure, respectively, indicating acquisition of approximately 2.5–4.7-fold taxotere resistance.

### Effects of protein kinase inhibitor on taxotere-induced cytotoxicity

**Figure 1:**
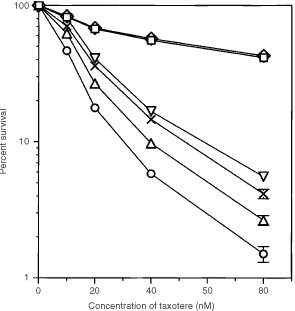
Effects of herbimycin A (HA) on the cytotoxicity of taxotere in parental HAG-1 and HAG/src3-1cells. Cells were treated with various concentrations of taxotere for 2 h and incubated for an additional 3 days, with or without HA. Cell survival was determined on day 7. Cytotoxicity curves of taxotere for HAG-1 (□) and HAG/src3-1 (○) without HA treatment. HAG-1 cells co-treated with taxotere and 100 ng ml^−1^ of HA (⋄). HAG/src3-1 cells co-treated with taxotere and 10 (▵), 50 (×) or 100 (▿) ng ml^−1^ of HA. The data represent the means from five independent experiments. *bars*, s.d. The s.d. for all points without error bars is less than 5%.

To determine whether p60^v-*src*^ PTK activity is required for sensitivity to taxotere in v-*src*-transfected cells, we studied the effect of HA on the taxotere sensitivity in parental HAG-1 and HAG/src3-1 cell lines. Combined treatment with taxotere and HA did not alter the sensitivity to taxotere in parental cells, but significantly reduced taxotere sensitivity in v-*src*-transfected cell line, in a dose-dependent manner, and sensitivity was reversed, if not completely, at the concentration of 100 ng ml^−1^ of HA (Figure 1

). To determine whether PI-3 kinase and/or PKC signal transduction pathways are involved in the mechanism of taxotere-induced sensitivity, we examined the effect of PKC inhibitor, H-7, and the effect of PI-3 kinase inhibitor, wortmannin, on taxotere cytotoxicity. Neither H-7 nor wortmannin at the non-toxic maximal concentration (20 nM) affected the cytotoxicity of taxotere in both parental HAG-1 and HAG/src3-1 cell lines (data not shown). These data indicate that taxotere sensitivity observed in HAG/src3-1 cells is partly induced by Src tyrosine kinase, but not by either PI-3 kinase or PKC.

### Dose-dependent effect of taxotere on Bcl-2 phosphorylation and inter-nucleosomal DNA fragmentation

**Figure 2:**
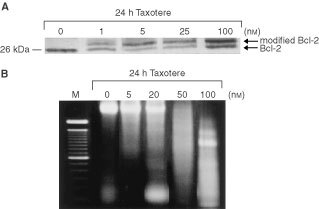
Dose-dependent effects of taxotere on Bcl-2 phosphorylation (**A**) and internucleosomal DNA fragmentation (**B**). (**A**) Western blot analysis of total proteins extracted from DMSO- or taxotere-treated HAG/src3-1 cells. The cells were treated either with vehicle solvent alone or with several taxotere concentrations (1–100 nM) for 24 h. After treatment, total cellular proteins were isolated as described in Materials and Methods. Equal amounts of protein were fractionated by 12% SDS–PAGE, transferred to nitrocellulose filter, and then blotted by monoclonal antibody against Bcl-2 protein. The arrows indicate the position of unphosphorylated and phosphorylated (modified) Bcl-2. Representative blots are shown. (**B**) Agarose electrophoresis of total DNA isolated from HAG/src3-1 cells following DMSO or taxotere exposure. The cells were treated either with vehicle solvent alone or with various concentrations of taxotere (5–100 nM) for 24 h and incubated for an additional 24 h in drug-free medium. Lane *M*, 100-bp DNA ladder.

The slower-migrating form of Bcl-2 was evident in HAG/src3-1 cells exposed to taxotere concentrations as low as 1 nM for 24 h, but not in DMSO-treated cells as a control, and a maximal effect was observed with a 100 nM taxotere concentration (Figure 2A

). This modified form of Bcl-2 has been shown to represent phosphorylated form of the Bcl-2 protein (Haldar *et al*, 1995). In parallel with Bcl-2 phosphorylation, taxotere-induced apoptosis, as determined by DNA fragmentation assay, was observed in HAG/src3-1 cells incubated with 100 nM taxotere for 24 h (Figure 2B). Agarose electrophoresis of DNA extracted from these cells showed a characteristic laddering pattern of oligonucleosomal DNA fragments resulting from internucleosomal chromatin cleavage by endogenous endonucleases.

### Time-course analysis of taxotere-induced Bcl-2 phosphorylation and inter-nucleosomal DNA fragmentation

**Figure 3:**
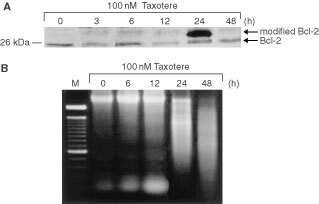
Kinetics of taxotere-induced Bcl-2 phosphorylation (**A**) and internucleosomal DNA fragmentation (**B**). The cells were exposed to 100 nM taxotere for 24-h and incubated in drug-free medium for indicated times. (**A**) Immunoblot of the total protein extract. Equivalent amounts of immunoprecipitates from HAG/src3-1 cells were subjected to 12% SDS–PAGE, followed by electroblotting to nitrocellulose, and then blotting by anti-Bcl-2 antibody. Arrows, unmodified and modified forms of Bcl-2. (**B**) Representative agarose gel of DNA isolated from HAG/src3-1 cells treated with 100 nM taxotere. Lane *M*, 100-bp DNA ladder.

Next we examined kinetics of Bcl-2 phosphorylation in HAG/src3-1 cells. The phosphorylated form of Bcl-2 reached a maximum with a 100 nM taxotere concentration at 24-h post-treatment, but declined drastically at 48 h (Figure 3A

). In accordance with Bcl-2 phosphorylation, the amount of oligonucleosomal DNA fragments increased with incubation time after taxotere treatment, and a maximal effect was observed with a 100 nM taxotere concentration at 48 h post-treatment (Figure 3B). At this time, only internucleosomal DNA fragments were present due to the complete apoptotic death of total cells. These data indicate that apoptotic events may be preceded by phosphorylation of Bcl-2. The observed decline in Bcl-2 phosphorylated form at 48 h post-treatment could be probably due to its degradation in the process of apoptotic cell death.

### Taxotere induces Bcl-2 phosphorylation and internucleosomal DNA fragmentation in v-*src*-transfected HAG/src3-1 cells

**Figure 4:**
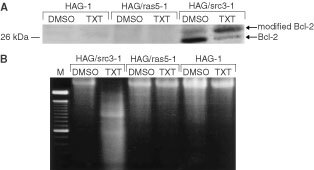
Taxotere induces phosphorylation of Bcl-2 and apoptosis only in v-*src-*transfected HAG/src3-1 cells. (**A**) Immunoblot analyses of total proteins extracted from DMSO-treated or taxotere-treated parental HAG-1, HAG/ras5-1 and HAG/src3-1 cell lines were carried out as described in Materials and Methods. All 3 cell lines were incubated with vehicle solvent alone or 100 nM taxotere for 24 h. Equal amounts of total protein extracts were electrophoresed on 12% SDS–PAGE, followed by transblotting to nitrocellulose membranes, and immunoblotted using a monoclonal antibody to Bcl-2. Arrows, unmodified and phosphorylated forms of Bcl-2. (**B**) Agarose gel electrophoresis of total DNA isolated from HAG/src3-1 cells treated with DMSO or taxotere. The cells were treated with vehicle solvent alone or 100 nM taxotere for 24 h, incubated for an additional 24 h in drug-free medium, and subjected to agarose gel electrophoresis. Lane *M*, 100-bp DNA ladder.

We tested whether there are any differences between Src and Ras signalling pathways in taxotere-induced apoptosis. The v-*src*-transfected HAG/src3-1 cells highly expressed Bcl-2, and phosphorylated form of Bcl-2 was evident by the treatment of 100 nM taxotere (Figure 4A

). By contrast, HAG-1 and HAG/ras5-1 slightly expressed Bcl-2, and failed to induce phosphorylation of Bcl-2 in the presence of taxotere (Figure 4A). Correspondingly, DNA fragmentation typical of apoptosis was observed only in Bcl-2-expressing HAG/src3-1 cells, but not in HAG-1 or HAG/ras5-1 cell lines (Figure 4B). These data indicate that v-Src, but not activated Ras, augments steady state levels of Bcl-2 expression and induces apoptosis through phosphorylation of Bcl-2.

### Effect of herbimycin A on the expression and phosphorylation status of Bcl-2 and internucleosomal DNA fragmentation

**Figure 5:**
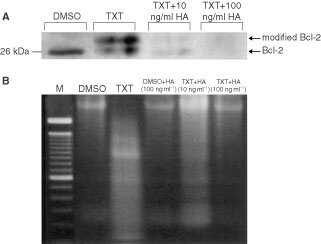
Effect of Src tyrosine kinase inhibitor, herbimycin A (HA) on the taxotere-induced phosphorylation status of Bcl-2 and internucleosomal DNA fragmentation. (**A**) Western blots of treated cell lysates were done as described in Materials and Methods. The HAG/src3-1 cells were treated with either DMSO (vehicle solvent), 100 nM taxotere, taxotere+10 ng/ml HA, or taxotere+100 ng ml^−1^ HA, for 24 h. Equivalent amounts of immunoprecipitates of anti-Bcl-2 antibody were separated by 12% SDS–PAGE, transferred on nitrocellulose sheet by electroblotting and then immunoblotted by monoclonal antibody against Bcl-2. The arrows indicate the position of unmodified and modified (phosphorylated) Bcl-2. (**B**) Representative agarose gel of DNA isolated from HAG/src3-1 cells. Cells were exposed to either DMSO±100 ng ml^−1^ HA, 100 nM taxotere, or taxotere±HA for 24 h, incubated for an additional 24 h in drug-free medium, and subjected to agarose gel electrophoresis. Lane *M*, 100-bp DNA ladder.

To determine whether Src PTK activity is required for taxotere-induced Bcl-2 phosphorylation and apoptosis, we examined the effect of HA on the expression and phosphorylation status of Bcl-2 and internucleosomal DNA fragmentation in HAG/src3-1 cells. Combined treatment with 100 nM taxotere and 10 ng ml^−1^ of HA markedly reduced expression of Bcl-2 and taxotere-induced phosphorylation of Bcl-2 (Figure 5A

), as well as internucleosomal DNA fragmentation (Figure 5B). At the concentration of 100 ng ml^−1^ of HA, the inhibition was almost complete, resulting in disappearance of both forms of Bcl-2 (Figure 5A). Similarly, taxotere-induced apoptosis, as determined by DNA fragmentation assay, was completely inhibited after co-treatment with 100 nM taxotere and 100 ng ml^−1^ of HA (Figure 5B). These findings indicate that Src tyrosine kinase activity is associated with Bcl-2 expression, its phosphorylation and apoptotic cell death.

### Intracellular taxotere content

To determine whether there are any differences of intracelluar taxotere content between taxotere-sensitive and taxotere-resistant cell lines, we directly measured cellular taxotere concentrations after treatment of cells with 55 nM taxotere for 2-h (IC_50_ values for parental HAG-1 cells). The resultant taxotere concentrations were 15.1±0.96 and 12.2±1.05 pM per 10^6^ parental HAG-1 and 10^6^ HAG/src3-1 cells, respectively, being not significantly different between these cell lines (*P*=0.20). These results indicate that intracellular drug accumulation does not play a role in taxotere-induced sensitivity in HAG/src3-1 cells.

## DISCUSSION

In the present study, we found that transfection of v-*src* renders human gall bladder epithelial HAG-1 cells sensitive to taxotere through augmentation of apoptotic cell death. A significant reduction of apoptosis was observed upon treatment with HA, a specific inhibitor of Src-family PTKs, suggesting that susceptibility to taxotere-induced apoptotic cell death is mediated by the activation of Src PTK. This is the first report demonstrating a direct association between taxotere-induced apoptosis and Src PTK activity. The intracellular taxotere contents were virtually identical between those cell lines, indicating that the intracellular drug accumulation does not explain the difference of taxotere sensitivity. v-Src, which has an intrinsic constitutively activated tyrosine kinase activity due to the lack of a negative regulatory domain, has been shown to phosphorylate a number of intracellular substrates on tyrosine residue (Brown and Cooper, 1996) and transduce signal throughout the cell to the nucleus. Among those signal transducers, Ras, which acts downstream of Src, may not be a cause of taxotere sensitivity, because activated Ras failed to induce taxotere sensitivity. Likewise, both PI-3 kinase and PKC pathways, which are activated directly or indirectly by v-Src through the association of SH2 and SH3 domains, appear not to be involved in the sensitivity mechanism, because inhibitors of these signal transduction pathways did not alter the sensitivity to taxotere. Although the data were not shown, Src also sensitized HAG-1 cells to taxol, another clinically useful taxane compound. Taxol induced apoptotic cell death at nearly 100-fold higher concentrations than taxotere. These data strongly suggest that the ability of activated Src to induce taxane sensitivity would be mediated by the augmentation of apoptosis through Src to downstream signal transduction pathways distinct from either Ras, PI-3 kinase, or PKC pathway.

Studies using taxanes have shown that Bcl-2 can be phosphorylated by taxanes at specific serine residues and that Bcl-2 phosphorylation is associated with loss of its anti-apoptotic function (Haldar *et al*, 1995; Srivastava *et al*, 1999). Therefore, we studied the effect of taxotere on phosphorylation status of Bcl-2. We have found that taxotere induces phosphorylation of Bcl-2 only in v-*src*-transfected HAG/src3-1 cells, but not in parental or c-H-*ras*-transfected HAG-1 cells. Consistent with previous observations, Bcl-2 phosphorylation is detected in the cells that underwent apoptotic cell death. According to the time course experiments, Bcl-2 phosphorylation precedes apoptotic events. Moreover, we demonstrated that HA abrogates taxotere-induced cell death and prevents Bcl-2 phosphorylation. These data suggest that Src tyrosine kinase augments taxotere-induced apoptosis presumably through phosphorylation of Bcl-2. At present, we do not know the precise mechanism whereby Src augments taxotere-induced phosphorylation of Bcl-2. However, several studies have suggested a role of Raf-1 serine/threonine kinase in the Bcl-2 phosphorylation following taxol treatment (Blagosklonny *et al*, 1996, 1997, 1999). Inhibition of RNA or protein synthesis prevents Raf-1 activation and Bcl-2 phosphorylation, suggesting that an intermediate protein(s) acts upstream of Raf-1 in this microtubule damage-activating pathway (Blagosklonny *et al*, 1997). Other study, however, has not confirmed this observation. Ibrado *et al* (1997) reported that taxol-induced apoptosis in HL-60 human myeloid leukaemia cells was not associated with activation of Raf-1. Our finding that activated Ras, which acts upstream of Raf-1, failed to induce taxotere sensitivity, suggest that involvement of Raf-1 kinase in taxotere-induced apoptotic signaling pathways is unlikely in v-*src*-transfected HAG-1 cells.

We have found that Bcl-2 is overexpressed only in v-*src*-transformed HAG/src3-1 cell line. This overexpression is reduced by Src tyrosine kinase inhibitor, suggesting a possibility that Src tyrosine kinase may enhance transcription of *bcl-2*. In this regard, we have recently shown that the signal transducer and activator of transcription 3 (STAT3) is constitutively activated in these HAG/src3-1 cells (Murakami *et al*, 1998). Therefore, we have hypothesized that STAT3 may promote the transcription of certain factors associated with Bcl-2 expression. Accordingly, it has been reported that decreased ability of STAT3 to bind DNA precedes decreased Bcl-2 expression and induction of apoptosis (Nielsen *et al*, 1999), indicating the involvement of STAT3 in the transcription of Bcl-2. The effect of dominant negative Stat3 on the Bcl-2 expression is currently under way. Moreover, the finding that taxotere induces apoptosis only in cells over-expressing Bcl-2 suggests that the apoptotic response of these cells to taxotere may depend on their Bcl-2 expression. Therefore, it will be important to determine whether other human neoplasms with Bcl-2 overexpression are also sensitive to the apoptotic action of taxotere.

In this study, we have demonstrated that v-*src* oncogene induces sensitivity to taxotere through Bcl-2 phosphorylation and apoptosis. By contrast, using the same cell line, we have recently shown that v-*src* induces cisplatin resistance through augmentation of the repair of CDDP-induced DNA cross-links (Masumoto *et al*, 1999). Therefore, taxane sensitivity and cisplatin resistance may be induced by the common signal transduction mechanism activated by Src tyrosine kinase. These data indicate that Src tyrosine kinase activity induces diverse effects on the chemosensitivity depending on the mechanism of action of anticancer agents. Several studies have demonstrated that Bcl-2 phosphorylation can be specifically induced by drugs that affect microtubule depolymerization or prevent microtubule assembly, whereas this effect is not seen by DNA damaging agents, such as cisplatin (Haldar *et al*, 1995, 1997). Therefore, apoptotic pathways induced by taxanes would be qualitatively different from those induced by cisplatin. There might be many signal transduction pathways of Src; some of them augments taxanes-induced apoptosis, and the others can prevent cell death by increasing the repair capacity of cisplatin-induced DNA damage. This hypothesis may explain the basis for the collateral sensitivity of cisplatin-resistant cell lines to taxol. Several studies have previously shown that cell lines resistant to cisplatin acquire taxol sensitivity (Perego *et al*, 1998; Judson *et al*, 1999). For instance, ovarian cell lines resistant to CDDP (C-13 and A2780CP) showed about 10-fold increase in sensitivity to taxol when compared to their respective parental cells, 2008 and A2780 (Judson *et al*, 1999). Similar observations have been noted in clinical studies which demonstrated that patients with cisplatin-resistant gynaecological cancers show an enhanced response to taxol (Woo *et al*, 1996).

In summary, human HAG-1 gall bladder adenocarcinoma cells are sensitized to apoptosis by taxotere through Bcl-2 phosphorylation as a consequence of activation of Src PTK. Therefore, it will be important to know the activation of Src before treatment, thus providing not only an opportunity to use therapeutic agents against more refined targeting, but also the advantage for selecting anticancer agents in individual cancers.
